# What does the general public understand about prevention and treatment of dementia? A systematic review of population-based surveys

**DOI:** 10.1371/journal.pone.0196085

**Published:** 2018-04-19

**Authors:** Monica Cations, Gorjana Radisic, Maria Crotty, Kate E. Laver

**Affiliations:** 1 Department of Rehabilitation, Aged and Extended Care, Flinders University, Adelaide, South Australia, Australia; 2 Cognitive Decline Partnership Centre, The University of Sydney, Sydney, New South Wales, Australia; University of Haifa, ISRAEL

## Abstract

**Objectives:**

To synthesise results of population surveys assessing knowledge and attitudes about prevention and treatment of dementia.

**Methods:**

MEDLINE, EMBASE, PsycINFO, and grey literature were searched for English language entries published between 2012 and May 2017. Survey questions were grouped using an inductive approach and responses were pooled.

**Results:**

Thirty-four eligible studies and four grey literature items were identified. Surveys were conducted in Europe, the US, Eastern Asia, Israel, and Australia. Nearly half of respondents agreed that dementia is a normal and non-preventable part of ageing, but belief in the potential for prevention may be improving over time. The role of cardiovascular risk factors was poorly understood overall. Less than half of respondents reported belief in the availability of a cure for dementia. The value of seeking treatment was highly endorsed.

**Conclusions:**

Results suggest that knowledge about the potential for dementia prevention and treatment remains poor but may be improving over time. Knowledge among those living in low- and middle-income countries are largely unknown, presenting challenges for the development of National action plans consistent with World Health Organization directives.

## Introduction

Approximately 47 million people worldwide are living with dementia (otherwise known as *major neurocognitive disorder*) and a new diagnosis is given every three seconds [1]. While research has not yet discovered a cure, there is accumulating evidence about the potential to prevent approximately one third of cases of dementia with management of risk factors such as poor educational attainment, hypertension, and depression [2]. In addition, both pharmacological and non-pharmacological treatments exist that can delay functional and cognitive decline [3,4], help to manage behaviour change [5,6], and improve wellbeing [7,8].

The recently adopted World Health Organization (WHO) Global Action Plan on Dementia urges all countries to implement campaigns to raise awareness about dementia [9]. The plan includes a global target that all member countries will have “at least one functioning public awareness campaign on dementia to foster a dementia-inclusive society by 2025” [9]. This focus reflects that population risk reduction and appropriate treatment for dementia rely on a contemporary understanding of these factors among the general public. Optimism about potential treatments can encourage early diagnosis, which allows for future planning and facilitates access to peer support, known to protect against psychological distress [10]. Understanding the modifiable risk factors for dementia may encourage preventative health behaviours in early and mid-life, ultimately reducing late-life incidence (and associated costs). However, misconceptions about dementia have been present for many years, including that dementia is a normal part of ageing and that there is no value in pursuing treatment [11]. These misconceptions have been noted to contribute to diagnostic delay as health professionals, people with symptoms and their families believe nothing can be done [12]. They also alleviate pressure on policy makers to devote funding to prevention and treatment services [13].

A systematic review of papers published to mid-2014 conducted by Cahill and colleagues [11] identified 40 studies of dementia literacy and reported only fair to moderate knowledge of dementia and a sparsity of evidence available in low- and middle-income countries. Since that time, major milestones in research and policy have occurred including the publication of hallmark reviews establishing the potential of dementia prevention [14], the proliferation of ‘dementia friendly community’ initiatives [15], and the establishment of dementia as a global health priority by WHO [9]. Public awareness campaigns have also become more prolific and have been delivered across a wider variety of platforms including social media [16]. However, many of these campaigns still focus on either the ‘catastrophic’ consequences of dementia or deliver overly simplistic or confusing messaging [17]. Whether such campaigns result in improved literacy about dementia prevention and treatment can inform future campaigns. This is particularly pertinent to the many low- and middle-income countries in the process of developing their first dementia action plans in response to the WHO directives. Previous reviews have not provided clear guidance about the key areas on which these campaigns should focus.

The aim of this review was to build on the work conducted by Cahill and colleagues by searching for more recent studies examining the population’s knowledge and understanding of dementia, and using this data to identify key target areas for public health focus We deliberately included only studies published in the past five years to represent contemporary thinking and explored whether there have been improvements in literacy over time. We endeavoured to understand whether the general public understand dementia as a preventable and treatable condition consistent with currently available evidence.

## Methods

The review protocol was registered on the PROSPERO database (CRD42017062286), and we report according to the Preferred Reporting Items for Systematic Reviews and Meta-Analyses (PRISMA) guidelines. A checklist of PRISMA items is presented in S1 Table.

### Data sources and searches

We searched MEDLINE, EMBASE and PsycINFO for English language studies published between 2012 and May 2017. We searched this timeframe only to report how closely contemporary views resemble recent available evidence about dementia prevention and treatment. The search strategy is available in S2 Table. Reference lists of all included studies were hand-searched for additional records. We also searched grey literature via a general internet search, Open Grey Europe, the Grey Literature Report, Web of Science, and report publications from Alzheimer's Disease International, national peak dementia organisations and the World Health Organisation.

### Eligibility criteria

Studies were included if they:

(a) Reported quantitative results of a survey (conducted via any method) of the general population;
(b) Included at least one question regarding views, knowledge, beliefs, or attitudes about prevention and/or treatment of dementia. Outcomes included knowledge about the existence of prevention or treatments strategies for dementia, as well as perceived efficacy of specific strategies, and;
(c) Were published from 2012 onwards.

Conference abstracts were included only if they provided quantitative data that could be used in the analysis.

Studies were excluded if they:

(a) Surveyed a specific population, such as people with dementia, carers of people with dementia, particular health professionals, or University students;
(b) Assessed attitudes to ageing in general or non-dementia conditions;
(c) Assessed fear of dementia, willingness to be screened for dementia (e.g. genetic testing), or stigma about dementia, unless the question directly related to prevention or treatment of dementia (e.g. “I would be screened for dementia because there are there are treatments to slow progression of the disease”);
(d) Reported results qualitatively only. Where a study reported mixed-methods results, only quantitative data was extracted and included;
(e) Reported results of a subset of participants from another, larger included study, or;
(f) Were published in a language other than English.

We requested raw data from authors where only synthesised results of a validated scale were reported (e.g. the Alzheimer’s Disease Knowledge Scale [18]).

### Study selection and data extraction

Study titles, abstracts, and full-texts were independently accessed and reviewed for eligibility by two authors. A data extraction form was developed and piloted with five studies before being finalised and used with the remaining studies. Two authors extracted the data, and this was then checked by a third author. Extracted data included authors, year of survey and publication, study aims, study design, sampling method, data collection method, participant details, and results.

### Quality assessment

Risk of bias was assessed at the study level. Two authors independently assessed the methodological quality of included studies using the ‘Qualitative descriptive studies’ section of the Mixed Methods Appraisal Tool (MMAT) [19]. The tool asks four yes/no/unclear questions: (1) is the sampling strategy relevant to address the quantitative research question? This criterion was met where the sampling method was clearly stated and appropriate to recruit a representative sample; (2) Is the sample representative of the population under study? This criterion was met where the inclusion and exclusion criteria were clearly explained, and participant characteristics were described; (3) Are measurements appropriate? Measures were considered appropriate where the question asked is clearly defined and asked similarly to all participants, and; (4) Is there an acceptable response rate? This criterion was satisfied with a response rate of 60 per cent or above. Quality assessment data were not used in data synthesis (see below) but were considered during interpretation of results to identify potential bias.

### Data synthesis

Two authors synthesised data by grouping similar responses into categories using an inductive approach modelled from McCullough et al [20]. Fixed responses (and the proportion of respondents endorsing them) were extracted and first grouped into six overarching categories that emerged from the data: general knowledge about dementia prevention, risk factors, protective factors, general knowledge about dementia treatment, pharmacological treatment, and non-pharmacological treatment. Data within these six categories were then organised into more specific groups where similar concepts were referenced. Groupings were checked by a third author. The specific items and categories used for prevention and treatment studies are available in S3 and S4 Tables respectively. Where studies reported a percentage of the sample agreeing with a statement, these were pooled and a median, interquartile range (IQR) and range were calculated. Answers were reverse coded where necessary. We plotted trends over time (by survey year) and compared continent responses (Europe, North America, Asia, Australia) to the four most commonly reported statements: ‘Dementia is a normal part of ageing’; ‘Dementia is not preventable’, ‘There is a cure for dementia’, and ‘Effective treatments exist for dementia’.

## Results

The search strategy identified 1364 unique records, and one additional citation was identified through hand-searching and grey literature searches. Following title and abstract screening, 1365 records were excluded due to non-relevance or not meeting inclusion criteria. One-hundred-and-one articles were accessed in full-text (S1 Fig). Of these, 33 met all inclusion criteria and were included in the review. Reasons for exclusion included that records did not discuss knowledge of treatment or prevention (*n* = 30), dementia (*n* = 3), or knowledge or attitudes (*n* = 6), were conference abstracts and provided insufficient detail (*n* = 7), included qualitative data only (*n* = 3), included data reported by a specific group (e.g. carers of people with dementia, medical professionals; *n* = 12), used the same data as another included record (*n* = 5), or did not provide raw scores (*n* = 2). The grey literature search revealed a conference abstract by Mi-Ra et al [21], a conference presentation by Dos Santos et al [22], and a research report by Dementia Australia [23]. Thirty-one studies reported data suitable for pooling. In total, the included studies surveyed 36,519 participants.

### Characteristics of included articles

Studies were published between 2012 and 2017 but reported on surveys conducted between 2008 and 2017 (Table 1). Study samples ranged from 50 to 3006 participants. Six of the eligible studies asked about the feasibility of treatments for dementia, seven asked about dementia prevention, and the remaining 19 included questions about both. Most surveys were conducted in Europe (*n* = 12), (UK, France, Germany, Poland, Spain, Portugal, Northern Ireland, Denmark, Italy). Eleven were conducted in the US, seven in Asia (China, South Korea, Singapore, Israel), and two in Australia. Africa, South and Central America, Canada, and Southern and Western Asia were not represented in any study. Six studies gathered views of specific ethnic groups within their country, including Black and Caribbean British communities [24], South Asian people living in the UK [25], Polish, Turkish, Danish, Pakistani people living in Denmark [26], or Chinese Americans [27–29]. Participants in most studies were randomly sampled via digit dialling or online access panels (*n* = 14) or convenience sampled via health and community services (*n* = 10).

**Table 1:** Included study characteristics.

First author, year	Treatment or prevention?	Country	Survey year	N	Sampling	Recruitment source	Assessment method	Response rate	Participant details
Almeling, 2014 [30]	Prevention	USA	2011	2100	Random	Access panel	Self-administered internet survey	39%	**Gender** 53% female; **Age** M = 50 (range n/r); **Ethnicity** 75% white, 10% African American
Dementia Australia, 2017 [23]	Both	Australia	2017	1049	Random	Access panel	Self-administered internet survey	n/r	**Gender** n/r; **Age** n/r, **Ethnicity** n/r
Ayalon, 2013 [31]	Prevention	USA	2010	1230	Random	Wave of prospective cohort study	Interviewer-administered face-to-face or telephone survey	100%	**Gender** 57.7% female; **Age** White 24.5% >75 years, Latino 8.8% >75 years, Black 23.4% >75 years (M, range n/r); **Ethnicity** 939 White, 120 Latino, 171 Black
Berwald, 2016 [24]	Treatment	England	2014	50	Purposeful	Community organisations	Face-to-face interview	n/r	**Gender** 60% female; **Age** M = 33 years (18–65); **Ethnicity** Black African 56%, Black Caribbean 28%, Black British 14%, Indo-Caribbean 2%
Blendon, 2012 [32]	Treatment	France, Germany, Poland, Spain, US	2011	2678	Random	Random digit dialling	Telephone interview	n/r	**Gender** n/r; **Age** n/r; **Ethnicity** n/r;
Bowes, 2012 [33]	Prevention	UK	2009	402	Purposeful	Employer organisations and online single interest discussion forums	Self-administered internet survey	Varies by item	**Gender** 50.7% female; **Age** 50–65 years (M n/r)**; Ethnicity** n/r
Breining, 2014 [34]	Both	France	2008	2013	Random	Random digit dialling	Telephone interview	10.90%	**Gender** 51.9% female; **Age** 11% 18–24 years, 15.4% 25–34 years, 29.9% 35–49 years, 22.7% 60–64 years, 21.1% 65 years or older (M n/r); **Ethnicity** n/r
Diamond, 2014 [35]	Both	USA	n/r	151	Convenience	Seminar attendees	Self-administered pen-and-paper survey	88%	**Gender** 72% female; **Age** 40–64 years (M n/r); **Ethnicity** Chinese
Dos Santos, 2015 [22]	Both	Portugal	2011	1476	Random	Random digit dialling	Telephone interview	n/r	**Gender** 79.7% female; **Age** M = 38.83 (18–94); **Ethnicity** n/r
Fowler, 2015 [36]	Treatment	USA	n/r	400	Purposeful	Health services	Telephone interview	n/r	**Gender** 66.9% female (St. Vincent Health) 73.8% female (Community Health Network); **Age** >65 years (M n/r); **Ethnicity** 78.1% White, 20.9% African American (St. Vincent Health) 96.7% White, 2.5% African American (Community Health Network)
Hailstone, 2017 [25]	Treatment	United Kingdom	n/r	51	Purposeful	Community organisations	Self-administered internet or pen-and-paper survey	n/r	**Gender** 66.7% female; **Age** M = 50.6 years (18–85); **Ethnicity** South Asian
Hudson, 2012 [37]	Both	United Kingdom	n/r	312	Convenience	Government institutions	Self-administered pen-and-paper survey	n/r	**Gender** 63% female; **Age** M Women = 53.3 years, M Men = 42.3 years (17–82); **Ethnicity** n/r
Leon, 2015 [38]	Both	France	2008 and 2013^b^	2008: 2013; 2013: 2509	Random	Random digit dialling	Telephone interview	n/r	**Gender** 52.2% female (2008), 52.4% female (2013); **Age** 2008: 50.1% 35–64 years, 21.2% >65 years; 2013: 51.5% 35–65 years, 21.8% 65 years and over (M n/r); **Ethnicity** n/r
Luck, 2012 [39]	Prevention	Germany	2011	1002	Random	Random digit dialling	Telephone interview	50.9%	**Gender** 50.3% female; **Age** M = 50.3 years (18–92)**; Ethnicity** n/r
Ludecke, 2016 [40]	Both	Germany	2012	1795	Random	Access panel	Self-administered pen-and-paper survey	78%	**Gender** 52% female; **Age** 31.4% 20–39 years, 37.8% 40–59 years, 30.8% 60–79 years (M n/r); **Ethnicity** n/r
McParland, 2012 [41]	Both	Northern Ireland	2010	1204	Random	Random digit dialling	Telephone interview	58%	**Gender** 52% female; **Age** 18–75 years (M n/r); **Ethnicity** 98% white
Mi-Ra, 2015 [21]	Both	South Korea	2014	926	n/r	n/r	Questionnaire	n/r	**Gender** n/r; **Age** n/r; **Ethnicity** n/r
Nguyen, 2016 [42]	Both	United States	n/r	102	Convenience	Community health fairs	Self-administered pen-and-paper survey	n/r	**Gender** 63.7% female; **Age** M = 50.56 years (range n/r); **Ethnicity** Vietnamese
Nielsen, 2016 [26]	Both	Denmark	2013	260	Mixed convenience / random	National registration system and snowball recruitment	Telephone interview	73%	**Gender** Danish 49% female, Polish 57% female, Turkish 35% female, Pakistani 39% female; **Age** >50 years, Danish M = 64.5, Polish M = 65.1, Turkish = 60.1, Pakistani = 58.3; **Ethnicity** Polish, Turkish, Danish, Pakistani
Park, 2016 [43]	Treatment	United States	n/r	626	Random	Access panel	Self-administered internet survey	16.7%	**Gender** 47% female; **Age** M = 74.3 years (65–92); **Ethnicity** White, not Hispanic 95.8%, Hispanic 0.5%, Black 1.9%, Asian or Pacific Islander 0.5%, American Indian, Eskimo, and Aleut 0.3%, Others 1.0%
Picco, 2016 [44]	Treatment	Singapore	2014–2015	3006	Random	National registration system	Face-to-face interview	71%	**Gender** 49.1% female; **Age** M = 40.9 years (18–65); **Ethnicity** Chinese 74.7%
Riva, 2012 [45]	Both	Italy	2008–2009	1111	Convenience	Hospital waiting rooms	Self-administered internet or pen-and-paper survey	n/r	**Gender** 63% female; **Age** 51% 18–40 years, 39% 41–64 years, 10% >65 years (M n/r); **Ethnicity** n/r
Roberts, 2014 [46]	Prevention	USA	2010	1641	Random	Wave of prospective cohort study	Interviewer-administered face-to-face or telephone survey	89.2%	**Gender** 53.6% female; **Age** M = 64.4 (range n/r); **Ethnicity** 8% Hispanic, 10.3% Black, 81.7% white
Seo, 2015 [47]	Both	South Korea	2011	2189	Convenience	Health services	Self-administered pen-and-paper survey	78.2%	**Gender:** 65.1% female; **Age:** 10–70+ years (M n/r); **Ethnicity:** n/r
Shinan-Altman, 2017 [48]	Prevention	Israel	n/r	236	Convenience	n/r	Face-to-face interview	n/r	**Gender** 49.4% female; **Age** M = 59 years (50–86); **Ethnicity** n/r
Smith, 2014 [49]	Prevention	Australia	2012	1003	Random	Random digit dialling	Telephone interview	58%	**Gender** 57.1% female; **Age** M = 47.6 (range n/r); **Ethnicity** n/r
Sites, 2016 [50]	Both	USA	2013	317	Random	Access panel	Self-administered survey (method n/r)	n/r	**Gender** 49% female; **Age** Median 49 years (M n/r); **Ethnicity** 80% White-non-Latino)
Sun, 2014 [29]	Both	USA	n/r	385	Purposeful	Community organisations	Face-to-face interview	n/r	**Gender** 64.2% female; **Age** M = 72.4 years (range n/r); **Ethnicity** Chinese American
Tan, 2012 [51]	Prevention	Singapore	n/r	338	Convenience	Hospital waiting rooms	Self-administered pen-and-paper survey	91.4%	**Gender:** 66.9% female; **Age:** 28.9% <65 years, 71.1% >65 years (M, range n/r)**; Ethnicity:** 88.1% Chinese
Woo, 2013 [28]	Both	United States	n/r	288	Convenience	n/r	Self-administered pen-and-paper survey	88.0%	**Gender** 67.7% female; **Age** 69.8% <65 years, 30.2% >65 years (M, range n/r); **Ethnicity** Chinese American
Yang, 2015 [52]	Both	China	2014	140	Convenience	Community organisations	Self-administered pen-and-paper survey	n/r	**Gender** 59.3% female; **Age** M = 56.19 years (20–75); **Ethnicity** n/r
Zeng, 2015 [53]	Both	China	2013	2000	Random	Direct approach at public places	Face-to-face interview	n/r	**Gender** 59.3% female; **Age** 35% 18–34 years, 53.8% 35–64 years, 11.2% >65 years (M, range n/r); **Ethnicity** n/r
Zheng, 2016 [27]	Both	USA	n/r	316	Convenience	Seminar attendees	Self-administered pen-and-paper survey	88.3	**Gender** 67.1% female; **Age** >55 years (M, range n/r); **Ethnicity** Chinese American

n/r = Not reported; M = mean

### Results of quality assessment

Results of the quality assessment are presented in S5 Table. Most studies were well-designed to gauge the views of the broad population of interest, and largely reported sample characteristics and results appropriately. All studies used standardised questions to gauge knowledge and attitudes and asked these consistently. However, only 17 reported their response rate and nine of these were below the ‘accepted’ threshold of 60 per cent.

### Knowledge about dementia prevention

**Fig 1:**
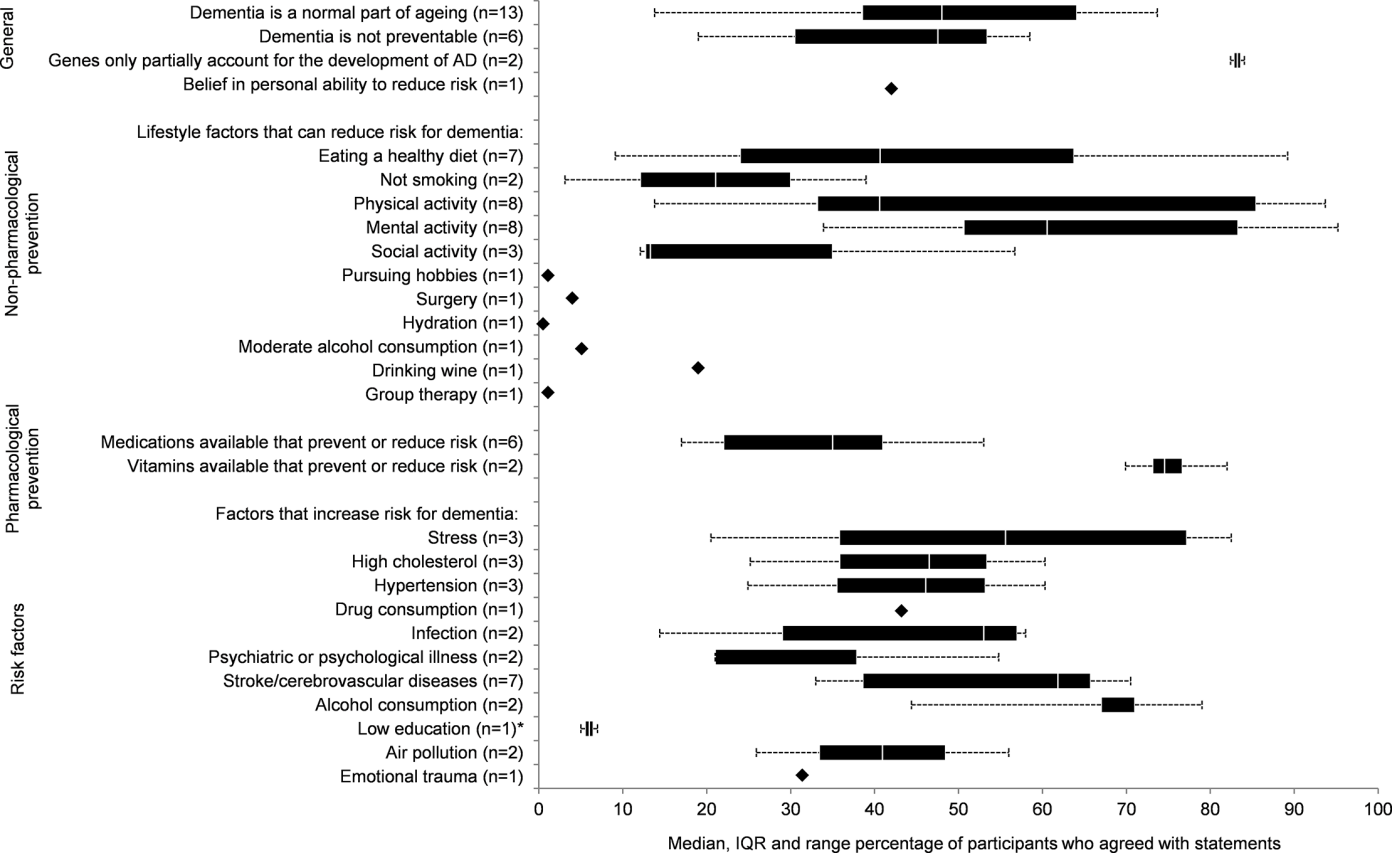
Synthesis of public knowledge and beliefs about prevention of dementia.

Twenty-six studies asked respondents about dementia prevention (Fig 1) [21–23,26,27,29–31,33–35,37–39,41,42,45–50,52–54]. Nearly half of respondents agreed that dementia is a normal part of ageing (from 13 studies; Median 48%, range 39–74%, *n* = 12,026) [23,27,28,35,38,41–43,45,47,51–53] and that dementia is not preventable (from six studies; Median 48%, range 19–59%, *n* = 9869) [21,38,47,49,53,55]. Consistent with this, one Australian study additionally reported that only 42 per cent of participants believed they could act to reduce their own risk (42%, *n* = 1003) [49]. However, two studies (one in the US and one in the UK) reported high levels of agreement that genetic factors only partially account for the development of dementia (Median 83%, range 83–84%, *n* = 629) [37,50].

Belief that specific non-genetic factors increase the risk for dementia was highest with alcohol consumption (Median 71%, range 67–88%, *n =* 1736) [22,26], stroke (Median 62%, range 33–71%, *n* = 4137) [22,26–28,35,42,47], stress (Median 56%, range 38–83%, *n* = 4347) [22,31,56], and infection (Median 53%, range 14–58%, *n* = 1736) [22,26]. Fewer than half of respondents believed that risk for dementia was associated with high cholesterol (Median 47%, range 25–60%, *n* = 1014) [29,37,50], hypertension (Median 46%, range 25–60%, *n* = 1014) [29,37,50], drug consumption (Median 43%, *n* = 1476) [22], air pollution (Median 41%, range 26–56%, *n* = 4013) [34,53], emotional trauma (Median 31%, *n* = 1476) [22], or psychiatric or psychological illness (Median 26%, range 21–55%, *n* = 4063) [22,45]. Despite its well-established relationship with dementia, only six per cent of respondents agreed that low education increased risk in the one study in which it was included (*n* = 1111) [45]. Dos Santos et al [22] reported that 25 per cent of respondents in Portugal believed that ‘use of medications’ could increase the risk for dementia, but did not specify to which medications this referred (*n* = 1476).

Most respondents in six studies did not believe that there are medications available to prevent or reduce the risk of dementia (Median 37%, range 17–53%, *n* = 6370) [29–31,37,55,56]. On the other hand, 75 per cent of participants in two US studies [31,46] believed vitamins are available to prevent or reduce risk for dementia (*n* = 2571). Most respondents in eight studies agreed that risk for dementia was reduced with mental activity (Median 61%, range 34–95%, *n* = 9313) [31,34,37,40,49,50,55,56]. However, when Bowes et al [33] specifically asked why respondents aged 50–65 years old participated in mental activities, very few reported ranked dementia risk reduction as their primary aim (1–8%). Belief in physical activity to reduce risk for dementia was moderate overall (Median = 41%, *n* = 11,966) but endorsement in individual studies ranged from 14 to 94 per cent [21,30,31,34,47,49,55,56]. Those with lower endorsement may be more representative as they tended to employ random digit dialling for recruitment [30,39] while those with higher endorsement were already involved in a prospective cohort study of ageing [31] or were recruited from health services [47]. All other non-pharmacological prevention strategies were poorly endorsed, including eating a healthy diet (Median 37%, range 9–89%, *n* = 10453) [26,30,31,34,49,55,56], not smoking (Median 21%, range 3–39%, *n* = 3016) [34,49], social activity (Median 13%, range 12–43%, *n* = 3481) [22,49,55], and moderate alcohol consumption (Median 5%, *n* = 1003) [49]. Three per cent of participants in Luck et al [39] agreed that ‘scientific research’ could reduce the risk for dementia but did not specify to what research this referred (*n* = 1002). Finally, Shinan-Altman et al [48] did not report raw scores and could not be pooled, but reported below average endorsement on a scale of one (strongly disagree) to five (strongly agree) that AD is attributable to risk factors (M = 2.47), psychological factors (M = 2.05), and immunity (M = 1.83).

### Knowledge about dementia treatment

**Fig 2:**
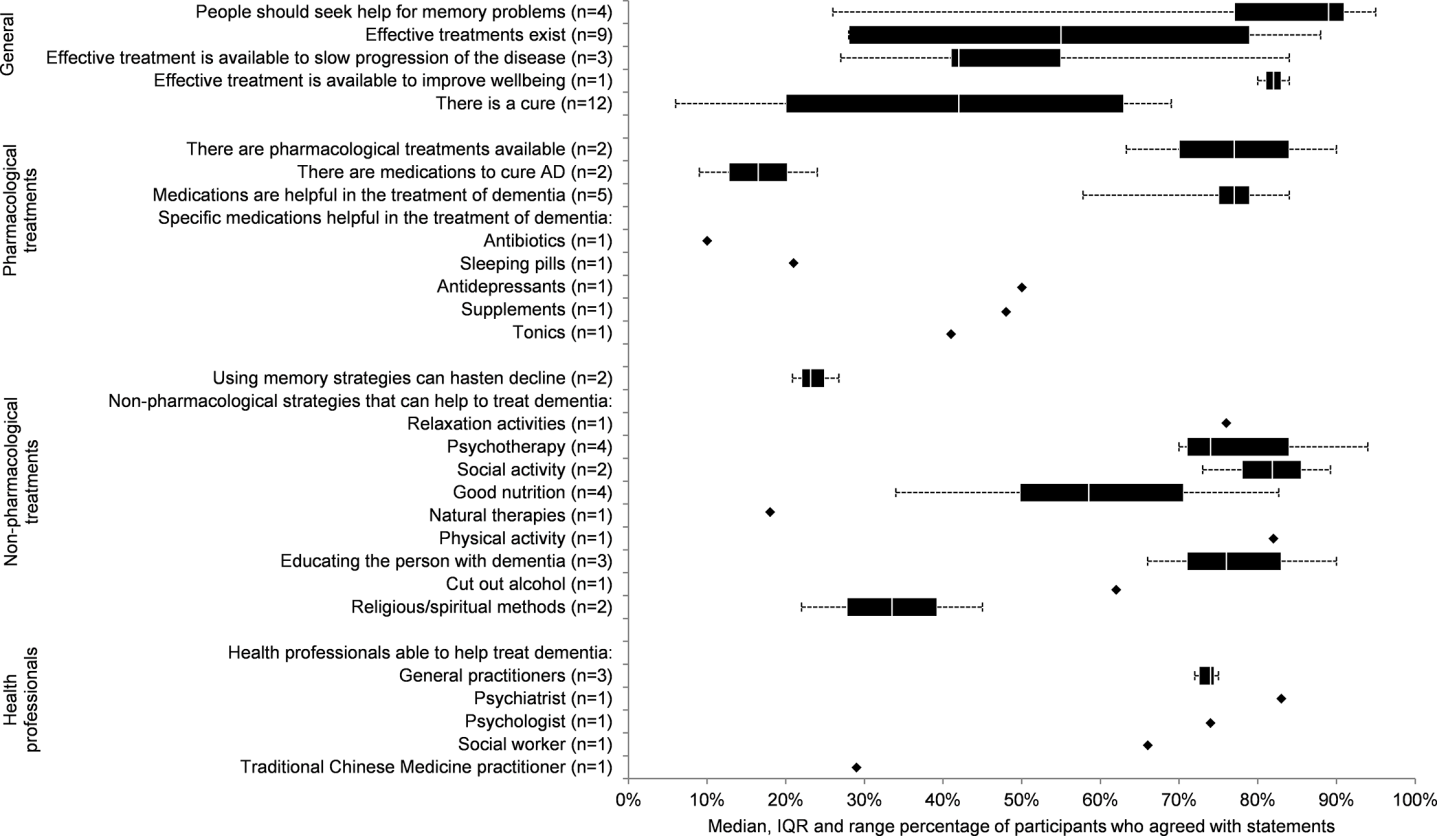
Synthesis of public knowledge and beliefs about treatments for dementia.

Twenty-five studies included questions about the efficacy of potential treatments for dementia (Fig 2) [21–28,32,34–38,40–42,44,45,47,50,52,53,57,58]. The most common question across studies concerned the availability of a cure, to which 42 per cent agreed (range 6–69%, *n =* 14,036) [21,26,29,32,34,37,41,44,47,52,53,57,58]. Two studies asked specifically about medications to cure dementia, but belief that these existed was very low (Median 17%, range 13–24%, *n* = 2421) [40,43].

Despite the general consensus that a cure was not available, there was a high level of agreement that people should seek help for memory problems (Median 89%, range 26–95%, *n* = 3794) [22,24,32,52]. This was accompanied by a generalised belief that ‘effective treatments exist’ (Median 55%, range 28–88%, *n* = 7846) though participants were less convinced that effective treatments exist to slow the progression of the disease (Median 42%, range 27–84%, *n* = 5617) [22,32,34]. There was a strong belief that an effective treatment is available to improve the wellbeing of people with dementia in one study with two cohorts (Median = 82%, range 80–84%, *n* = 4522) [38].

Two studies reported that participants were mostly aware that pharmacological treatments are available for dementia (Median 77%, range 63–90%, *n =* 2587) [22,45], and five studies reported a general belief that these treatments are effective (Median = 77%, range = 58–84%, *n =* 7960) [21,41,44,45]. However, when Picco et al [44] asked about specific medications, belief in their efficacy ranged from 10% for antibiotics to 50% for antidepressants. Belief in the efficacy of alternate therapies was moderate overall, including 41% endorsing tonics and 48% endorsing supplements. No studies asked about the utility of acetylcholinesterase inhibitors.

Seven studies included questions about the potential for non-pharmacological treatments to be helpful in the treatment of dementia [21,24,29,37,44,50,52]. Of these, social activity [44,52] and physical activity [44] were considered most beneficial overall and were both endorsed by a median of 82% of respondents (social activity range 72–91%, *n* = 3146; physical activity *n* = 3006), followed by relaxation activities (Median 76%, *n* = 3006) [44], in-person psychotherapy or counselling (Median 74%, range 70–90%, *n* = 4330) [44,50,58,59], cutting out alcohol (Median 62%, *n* = 3006) [44], and eating a healthy diet (Median 59%, range 34–78%, *n* = 4330). Picco et al [44] additionally noted that most respondents endorsed seeking help from close family (Median 84%) and friends (Median 78%), and yoga or meditation (Median 68%). Religious or spiritual methods (Median 34%, range 22–45%, *n* = 3056) [24,44] and natural therapies (Median 18%, *n* = 50) [24] were endorsed by fewer than half of respondents included. The value of educating the person with dementia about their illness was recognised in two studies both conducted in Asia (Median 76%, range 65–89%, *n* = 5670) [21,44].

Two studies asked respondents about specific health professionals who could be helpful in the treatment of dementia [24,44]. Both reported a high level of belief that a general practitioner can be helpful (Median 73%, range 73–74%, *n* = 3056). Picco et al [44] additionally reported a moderate to high level of endorsement for psychiatrists (Median 83%), psychologists (Median 74%), and social workers (Median 66%), but not for traditional Chinese medicine practitioners (Median 29%), among the general public in Singapore. Hailstone et al [25] reported results on a scale from 1 (strongly disagree) to 7 (strongly agree) and so could not be pooled, but participants overall agreed that they would want to see their doctor if they had memory problems, that seeking help from their doctor would be beneficial, valuable, and good, and that their doctor would be able to provide treatments to help with memory problems.

### Trends by time, location

**Fig 3:**
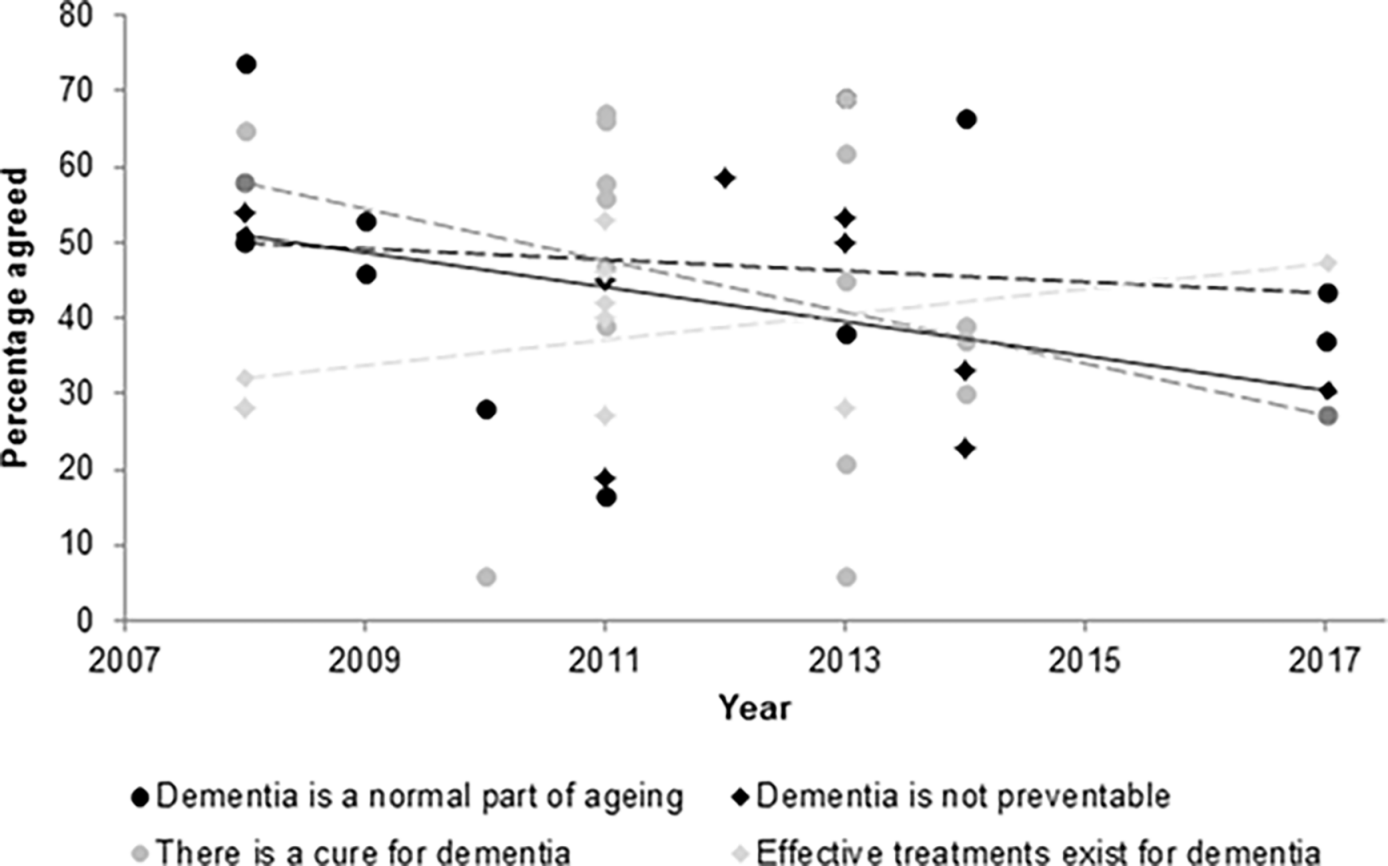
Trends by time.

Survey administration year (where reported) was plotted against endorsement of the four most commonly included statements across studies (Fig 3). A downward trend was noted in belief that there is a cure for dementia, while belief that effective treatments exist appears to have increased over time. The understanding that dementia is a preventable disease also appears to be increasing. However, belief that dementia is a normal part of ageing has remained relatively steady over the eight-year period.

Pooled responses to these same questions were stratified by continent (Table 2). No striking patterns emerged, and continents were relatively homogenous in their knowledge. European and American respondents were more likely than Asians and Australians to believe dementia is a normal part of ageing. Six studies gathered views of specific ethnic groups within high-income countries, but results were unremarkable relative to other studies.

**Table 2:** Trends by continent.

	Pooled median (range)			
	Europe	US	Asia	Australia
Dementia is a normal part of ageing	53% (28–74)	53% (14–72)	43% (16–66)	39% (n/a)
	*n* = 6837	*n* = 1483	*n* = 4667	*n* = 1049
Dementia is not preventable	53% (45–54)	n/a	28% (19–50)	59% (n/a)
	*n =* 5524		*n =* 5115	*n* = 1003
There is a cure for dementia	56% (6–69)	38% (16–64)	38% (13–62)	n/a
	*n* = 5828	*n* = 1100	*n* = 8261	
Effective treatments exist for dementia	40% (27–88)	59% (46–81)	n/a	n/a
	*n* = 10100	*n* = 1346		

n/a = Not available

## Discussion

The aim of this review was to compare the general public’s understanding of dementia as a preventable and treatable condition to contemporary scientific evidence. Thirty-three eligible surveys of the general population were identified, conducted predominantly in Europe and the US and occasionally in Eastern Asia and Australia. Results suggest that knowledge about the potential for dementia risk reduction and treatment of symptoms remains poor but may be improving over time. Knowledge and attitudes of those living in low- and middle-income countries are largely unknown.

### Main findings

Twenty-five of the studies included in this review were published since a narrative review of public knowledge and attitudes about dementia was published in 2014 [11]. Results of that review were strikingly similar to those reported here, including the common misconception that dementia is a normal part of ageing and is not preventable. As authors of the earlier review point out, these misconceptions have been documented for decades even among health professionals who diagnose and treat dementia [60]. There were some positive signs in terms of improvements in awareness over time, particularly among ethnic minority groups living in high-income countries. Cognitive leisure activities in particular appear to be well understood as good candidates for dementia prevention or delay, consistent with some evidence that they can delay the onset of dementia [2]. On the other hand, the importance of formal educational attainment and management of cardiovascular were acknowledged by fewer than half of respondents who were asked about them.

Despite the generalised understanding that dementia is usually a terminal condition without an available cure belief in the value of seeking treatment was high in almost all studies and suggests a positive shift in attitudes away from fatalistic beliefs that have acted as a barrier to help seeking in the past [61]. It was noted, however, that the perceived value in treatment lay mostly in its potential to support wellbeing rather than slow the progression of symptoms. This is contrary to evidence that both pharmacological and non-pharmacological methods can delay functional and cognitive decline [3,62]. The general public was positive overall about the value of practicing healthy behaviours after a diagnosis of dementia. Whether these strategies were believed to limit progression of disease or were simply viewed as valuable health behaviours more generally (as has been demonstrated in the past [63]) was not explored and is an important avenue for future research

There were large geographic and cultural gaps in the available literature. Low-income countries were not represented, and populous countries with rapidly ageing populations were notably missing (e.g. Japan, India). Given the socio-economic homogeneity of countries where data is available, it is not surprising that no major trends emerged in comparisons by continent. The lack of data in low- and middle-income countries was also noted by Cahill and colleagues in their earlier review [11]. That little progress has been made to fill this gap in the subsequent years is troubling particularly because most people with dementia live in low- and middle-income regions [1] and because structural barriers to awareness and help-seeking are more common in those regions [13]. Gathering a baseline understanding of public knowledge and attitudes in low- and middle-income regions is essential for development of targeted public awareness campaigns.

### Strengths and limitations of review and included studies

This review benefited from a robust search strategy that was complemented by a thorough examination of the grey literature. It is the first to inductively pool similar responses and present findings visually for ease of interpretation and analysis of trends over time. Nonetheless, the results should be interpreted in the context of important limitations. First, studies published in languages other English were excluded and this may have precluded the representation of many regions and cultural groups. This is particularly important given how little is known about knowledge and attitudes in non-English speaking regions, and the structural barriers to awareness raising that exist in these regions. Second, nearly half of the included studies did not report their response rate. Of those that did (*n* = 17), only eight reported a rate above 60 per cent. Non-response bias is possible and the reported views may overestimate general population knowledge. On the other hand, publication bias and selective reporting within studies is possible if authors choose to publish findings only where knowledge is ‘remarkably’ low. This is less likely given the variety in knowledge levels reported by the included studies, but some bias may still exist. Third, synthesising the data via quantitative meta-analysis was precluded by heterogeneity of the questions posed. Pooling ‘like’ themes was considered more appropriate in this case. Finally, an analysis of themes from qualitative studies in subsequent reviews will add depth to our understanding of public awareness, including the socio-political factors that allow misconceptions to persist.

### Implications

Results of this study suggest several key areas of need in general public dementia literacy. The view that dementia is a normal part of ageing with few treatment options is a demonstrated barrier to both preventive health behaviours and to help-seeking and diagnosis in the event that symptoms emerge [12]. Stigmatisation occurs in the absence of accurate understanding, and contributes to social isolation and emotional distress for people with dementia and their carers [13].

While the proliferation of public awareness campaigns and dementia-friendly community initiatives in high-income countries appears to be having a positive impact, gaps in knowledge remain and present key target areas for future campaigns. First, a significant underestimation of the importance of non-genetic cognitive and cardiovascular risk factors is evident and is not helped by confusing messaging about what is and is not harmful [17]. Dementia is a complex syndrome for which the impact of risk factors can vary depending on the type of dementia, timing of exposure, confluence of risks, and pre-existing genetic susceptibilities [6]. The quality of evidence regarding individual risks varies, and conflicting findings regularly emerge. In this context, researchers have a responsibility to disseminate their findings to the public in ways that do not promote misunderstandings, including working in partnership with the mainstream media. In the meantime, simple messaging about the net benefit of healthy behaviours over the life course may be most beneficial.

Second, there appears to be a misconception that available treatments are useful only for maintaining the wellbeing of people with dementia and are not able to slow progression of disease. This may be related to the focus in public awareness campaigns on the serious consequences of dementia. While ‘fear appeal’ campaigns can promote investments in research and care [64], they also (by nature) promote fear of the illness. This messaging must be complemented by evidence-based campaigns emphasising the value of seeking a diagnosis and treatment.

Third, the public tended to endorse poorly supported risk reduction strategies (like vitamins) over more powerful but also more effortful strategies (like exercise). Promotion of realistic risk reduction messages necessitates debunking less relevant strategies to reduce noise. Finally, the misconception that dementia is a normal part of ageing is persisting despite decades of public health efforts contrasting this message. New strategies are clearly required to address this.

Policy-makers in low and middle-income countries, especially those developing their first dementia action plans, will benefit from a better understanding of the barriers to knowledge in their countries and cost-effective methods to overcome these. The potential reach of public awareness campaigns is ever-increasing as technology makes information more accessible. At the same time, the media landscape is crowded and public health agencies must compete for attention [65]. The WHO Global Action Plan [9] advocates for both national and local public health campaigns that are community- and culture-specific and developed in consultation with people living with dementia and their carers. Educating children and young people may have particular benefits, as this approach can foster intergenerational solidarity and prepare a future generation of informal carers [13]. The introduction of alternative terminology (*neurocognitive disorders*) in the most recent iteration of the Diagnostic and Statistical Manual for Mental Disorders [66] was intended to reduce the stigma associated with ‘dementia’ (a term meaning ‘mad’ or ‘insane’ in Latin). Increasing use of this terminology may help to correct long-held misconceptions about dementia and frame a new understanding of the condition among the general public. Most importantly, creative messaging and methods of delivery must be paired with a supportive environment that enables the public to take the action advocated in the campaign [65]. Without sustainable infrastructure to facilitate risk reduction and help seeking, the benefits of improving public awareness will be stifled.

## Supporting information

S1 Fig PRISMA flowchart describing the process of study selection. (DOCX)

S1 Table PRISMA checklist. (DOCX)

S2 Table OVID search strategy (Medline, EMBASE, PsycINFO). (DOCX)

S3 Table Prevention results. M = Mean *Reverse scores used for pooling.
(DOCX)

S4 Table Treatment results. M = Mean *Reverse scores used for pooling ^a^59% ‘disagree’ or ‘strongly disagree’, 20% ‘don’t know’ ^b^91% disagree, 2% uncertain.
(DOCX)

S5 Table Results of quality assessment of peer-reviewed journal articles. Y = Yes, N = No, U = Unclear.
(DOCX)
