# Supplementary material for: What does the general public understand about prevention and treatment of dementia? A systematic review of population-based surveys
Source: PLoS One. 2018 Apr 19;13(4):e0196085. doi: 10.1371/journal.pone.0196085 (PMC5908164; doi:10.1371/journal.pone.0196085)
Supplement: S1 Fig — (DOCX) [file pone.0196085.s001.docx]

Studies included in qualitative synthesis
(*n*=33)

## Included

Studies included in quantitative synthesis (pooling)
(*n*=31)

Records identified through database searching
(*n*=2209)

## Screening

## Eligibility

## Identification

Additional records identified through other sources
(*n*=1)

Records after duplicates removed
(*n*=1365)

Record titles and abstracts screened
(*n*=1365)

Records excluded due to non-relevance or not meeting inclusion criteria
(*n*=1264)

Full-text articles assessed for eligibility
(*n*=101)

30 did not discuss knowledge of treatments or prevention

7 were conference abstracts with insufficient detail

3 were qualitative studies

12 surveyed a specific group (not general population)

3 were not about dementia

6 did not assess knowledge or attitudes

5 used the same sample/data as another paper

2 did not provide raw scores

Figure S1. PRISMA flowchart describing the process of study selection
