# Supplementary material for: What does the general public understand about prevention and treatment of dementia? A systematic review of population-based surveys
Source: PLoS One. 2018 Apr 19;13(4):e0196085. doi: 10.1371/journal.pone.0196085 (PMC5908164; doi:10.1371/journal.pone.0196085)
Supplement: S2 Table — (DOCX) [file pone.0196085.s003.docx]

Table S2. OVID Search strategy (Medline, EMBASE, PsycINFO)

| Number | Search term |
| --- | --- |
| 1 | Dementia/ or Alzheimer disease/ or dementia, vascular/ or cadasil/ or dementia, multi-infarct/ or frontotemporal lobar degeneration/ or frontotemporal dementia/ or “pick disease of the brain”/ or primary progressive nonfluent aphasia/ or lewy body disease/ |
| 2 | (dementia or Alzheimer* or lewy bod* or cadadsil or pick* or frontotemporal).tw,kw |
| 3 | Or/1-2 |
| 4 | Public opinion/ |
| 5 | Attitude to health/ or health knowledge, attitudes, practice/ or stigma/ |
| 6 | ((lay or public or general or communit* or people* or person*) adj4 (opinion* or knowledge* or belief* or perceive* or attitude* or experience* or understanding)).tw,kw. |
| 7 | Stigma*.tw,kw. |
| 8 | Or/4-7 |
| 9 | Cross-sectional studies/ |
| 10 | Cross-section*.tw,kw. |
| 11 | ((population* or nation* or countr*) adj3 (study or studies or interview*)).tw,kw. |
| 12 | Exp “Surveys and questionnaires”/ |
| 13 | (survey* or questionnaire* or vignette*).tw,kw. |
| 14 | Or/9-13 |
| 15 | 3 and 8 and 14 |
| 16 | Limit 15 to (English language and yr=“2012-Current”) |
