# Supplementary material for: What does the general public understand about prevention and treatment of dementia? A systematic review of population-based surveys
Source: PLoS One. 2018 Apr 19;13(4):e0196085. doi: 10.1371/journal.pone.0196085 (PMC5908164; doi:10.1371/journal.pone.0196085)
Supplement: S3 Table — M = Mean *Reverse scores used for pooling. (DOCX) [file pone.0196085.s004.docx]

Table S3. Prevention results.

| Study first author, year | Phrase | % yes/agreed |
| --- | --- | --- |
| PREVENTION GENERAL | | |
| *Dementia is a normal part of ageing* | | |
| Dementia Australia, 2017 | Dementia is not a normal part of ageing | 61%* |
| Diamond, 2014 | Dementia is forgetfulness due to ageing. Everyone will have dementia with advancing age | 43% |
| Leon, 2015 | It is normal to suffer memory loss as you get older   - 2008 cohort - 2012 cohort | 73.7%  69.1% |
| McParland, 2012 | Dementia is part of the normal process of ageing | 28% |
| Nguyen, 2016 | Dementia is forgetfulness due to ageing; everyone will have dementia with advancing age | 53% |
| Park, 2016 | Alzheimer’s disease is a normal process of ageing | 13.8% |
| Riva, 2012 | Do you think that dementia is normal with ageing?   - Caregivers - Non-caregivers | 46%  53% |
| Seo, 2015 | Everyone develops dementia when he or she becomes old | 16.4% |
| Tan, 2012 | Dementia is a normal part of ageing such that all older people develop dementia as they age | 48.1% |
| Woo, 2013 | Dementia is forgetfulness due to aging; everyone will have dementia with advancing age | 71.5% |
| Yang, 2015 | Alzheimer’s disease is caused by normal ageing, and everyone will suffer from it when one gets old | 66.4% |
| Zeng, 2015 | It is normal to get dementia when you get old | 38.1% |
| Zheng, 2016 | Dementia is forgetfulness due to ageing. Everyone will have dementia with advancing age | 61.8% |
| *Dementia is not preventable* | | |
| Leon 2015 | Nothing can be done to avoid getting Alzheimer’s disease   - 2008 cohort - 2013 cohort | 53.8%  53.3% |
| Luck 2012 | Dementia can be prevented | 55%* |
| Mi-Ra 2015 | There is no way to prevent dementia   - Male - Female | 22.9%  33% |
| Seo 2015 | There is no way to prevent dementia | 19% |
| Smith 2014 | Strongly believe the risk for dementia can be reduced | 41.5%* |
| Zeng 2015 | We cannot prevent dementia now | 50.1% |
| *Genes only partially account for the development of dementia* | | |
| Hudson 2012 | Genes can only partially account for the development of Alzheimer’s disease | 82.4% |
| Stites 2016 | Genes can only partially account for the development of Alzheimer’s disease | 18.4% |
| *Belief in personal ability to reduce risk* | | |
| Smith 2014 | I am confident that I can take action to reduce my risk | 41.5% |
| NON-PHARMACOLOGICAL PREVENTION | | |
| *Eating a healthy diet can reduce risk for dementia* | | |
| Almeling 2014 | To reduce risk, very likely to eat healthily | 24% |
| Ayalon 2013 | Healthy diet reduces the risk for developing Alzheimer’s disease   - White - Black - Latino | 89.2%  84.1%  86.2% |
| Breining 2014 | Regularly eating fruits and vegetables prevents dementia | 57% |
| Luck 2012 | Healthy diet and lifestyle can prevent dementia | 9.1% |
| Nielsen 2016 | Diet can cause dementia   - Danish - Polish - Turkish - Pakistani | 24%  54%  37%  32% |
| Roberts 2014 | Eating a healthy diet is a very effective protective factor | 44.3% |
| Smith 2014 | Healthy diet is beneficial for dementia risk reduction | 23.3% |
| *Not smoking can reduce risk for dementia* | | |
| Breining 2014 | Not smoking prevents dementia | 39% |
| Smith 2014 | Not smoking is beneficial for dementia risk reduction | 3.1% |
| *Physical activity can reduce risk for dementia* | | |
| Almeling 2014 | To reduce risk, very likely to exercise | 21% |
| Ayalon 2013 | Physical activity reduces the risk for developing Alzheimer’s disease   - White - Black - Latino | 90.0%  83.6%  87.3% |
| Breining 2014 | Regular physical exercise or sports prevents dementia | 68% |
| Luck 2012 | Sport/exercise/physical fitness can prevent dementia | 13.8% |
| Mi-Ra 2015 | Regular exercise reduces the risk of dementia   - Male - Female | 37.7%  35.1% |
| Roberts 2014 | Keeping physically active is a very effective protective factor | 40.6% |
| Seo 2015 | Regular exercise reduces the risk of dementia | 93.7% |
| Smith 2014 | Physical activity is beneficial for dementia risk reduction | 31.3% |
| *Mental activity can reduce risk for dementia* | | |
| Ayalon 2013 | Mental activity reduces the risk for developing Alzheimer’s disease   - White - Black - Latino | 95.2%  84.4%  88.2% |
| Breining 2014 | Doing crosswords or memory games prevents dementia | 80% |
| Hudson 2012 | It has been scientifically proven that mental exercise can prevent a person from getting Alzheimer’s disease | 59.7% |
| Luck 2012 | Mental activity can prevent dementia | 33.9% |
| Ludecke 2016 | It has been scientifically proven that mental exercise can prevent a person from getting Alzheimer’s disease | 48.5% |
| Roberts 2014 | Keeping mentally active is a very effective protective factor | 61.4% |
| Smith 2014 | Mental activity is beneficial for dementia risk reduction | 57.1% |
| Stites 2016 | It has been scientifically proven that mental exercise can prevent a person from getting Alzheimer’s disease | 43.4% |
| *Social contact can reduce risk for dementia* | | |
| Dos Santos 2015 | Social isolation causes dementia | 43.3%* |
| Luck 2012 | Social contacts/avoidance of social isolation can prevent dementia | 13.8% |
| Smith 2014 | Social activity is beneficial for dementia risk reduction | 12.1% |
| *Pursuing hobbies can reduce risk for dementia* | | |
| Luck 2012 | Pursuing hobbies can prevent dementia | 1.1% |
| *Surgery can reduce risk for dementia* | | |
| Almeling 2014 | To reduce risk, very likely to have surgery | 4% |
| *Hydration can reduce risk for dementia* | | |
| Luck 2012 | Hydration can prevent dementia | 0.5% |
| *Moderate alcohol consumption can reduce risk for dementia* | | |
| Smith 2014 | Moderate alcohol is beneficial for dementia risk reduction | 5.1% |
| *Drinking wine can reduce risk for dementia* | | |
| Breining 2014 | Drinking wine prevents dementia | 19% |
| *Group therapy can reduce risk for dementia* | | |
| Luck 2012 | Group therapy can prevent dementia | 1.1% |
| PHARMACOLOGICAL PREVENTION | | |
| *Medications are available that prevent or reduce risk for dementia* | | |
| Almeling 2014 | To reduce risk, very likely to take medication | 17% |
| Ayalon 2013 | Prescription drugs that prevent Alzheimer’s disease are available   - White - Black - Latino | 34.6%  53.3%  48.4% |
| Hudson 2012 | Prescription drugs that prevent Alzheimer’s disease are available | 23.2% |
| Luck 2012 | Medication can prevent dementia | 18.4% |
| Roberts 2014 | Prescription drugs that prevent Alzheimer’s disease are available | 39.1% |
| Sun 2014 | Prescription drugs that prevent Alzheimer’s disease are available | 36.1% |
| *Vitamins are available to prevent or reduce risk* | | |
| Ayalon 2013 | Vitamins reduce the risk for developing Alzheimer’s disease   - White - Black - Latino | 74.2%  74.9%  82.0% |
| Roberts 2014 | Taking vitamins is a very effective protective factor | 20.5% |
| RISK FACTORS | | |
| *Stress increases the risk for dementia* | | |
| Ayalon 2013 | Stress increases the risk for developing Alzheimer’s disease (very important or somewhat important)   - White - Black - Latino | 55.6%  77.2%  82.5% |
| Dos Santos 2015 | Stress causes dementia | 35.8% |
| Roberts 2014 | Stress is a very important risk factor for Alzheimer’s disease | 20.5% |
| *High cholesterol increases the risk for dementia* | | |
| Hudson 2012 | Having high cholesterol may increase a person's risk of developing Alzheimer’s disease | 25.2% |
| Stites 2016 | Having high cholesterol may increase a person’s risk of developing Alzheimer’s disease | 46.5% |
| Sun 2014 | Having high cholesterol may increase a person’s risk of developing Alzheimer’s disease | 60.3% |
| *Hypertension increases the risk for dementia* | | |
| Hudson 2012 | Having high blood pressure may increase a person’s risk of developing Alzheimer’s disease | 24.9% |
| Stites 2016 | Having high blood pressure may increase a person’s risk of developing Alzheimer’s disease | 46.1% |
| Sun 2014 | Having high blood pressure may increase a person’s risk of developing Alzheimer’s disease | 60% |
| *Drug consumption increases the risk for dementia* | | |
| Dos Santos 2015 | Drug consumption causes dementia | 43.2% |
| *Infection increases the risk for dementia* | | |
| Dos Santos 2015 | Infection causes dementia | 14.4% |
| Nielsen 2016 | Infection causes dementia   - Danish - Polish - Turkish - Pakistani | 29%  57%  53%  58% |
| *Psychiatric or psychological illness increases the risk for dementia* | | |
| Dos Santos 2015 | Psychiatric disease causes dementia | 54.8% |
| Riva 2012 | Depression is a risk factor for Alzheimer’s disease   - Caregivers - Non-caregivers | 21%  21% |
| *Stroke/cerebrovascular disease increases the risk for dementia* | | |
| Dos Santos 2015 | Stroke causes dementia | 44.9% |
| Diamond 2014 | Some types of dementia are caused by cerebrovascular disease | 36.5% |
| Nguyen 2016 | Some types of dementia are caused by cerebrovascular disease | 64.7% |
| Nielsen 2016 | Stroke can cause dementia   - Danish - Polish - Turkish - Pakistani | 67%  66%  59%  65% |
| Seo 2015 | Stroke may lead to dementia | 70.5% |
| Woo 2013 | Some types of dementia are caused by cerebrovascular disease | 33% |
| Zheng 2016 | Some types of dementia are caused by cerebrovascular disease | 33% |
| *Alcohol consumption increases the risk for dementia* | | |
| Dos Santos 2015 | Alcohol consumption causes dementia | 44.4% |
| Nielsen 2016 | Alcohol can cause dementia   - Danish - Polish - Turkish - Pakistani | 71%  79%  67%  71% |
| *Low education increases the risk for dementia* | | |
| Riva 2012 | Low education is a risk factor for Alzheimer’s disease   - Caregivers - Non-caregivers | 7%  5% |
| *Air pollution increases the risk for dementia* | | |
| Breining 2014 | A non-polluted environment prevents dementia | 44%* |
| Zeng 2015 | Air pollution can cause Alzheimer’s disease | 25.9% |
| *Emotional trauma increases the risk for dementia* | | |
| Dos Santos 2015 | Emotional trauma causes dementia | 31.4% |
| NOT POOLED | | |
| Bowes 2012 | Primary reason for engaging in activity is to prevent onset of dementia:   - Reading - Writing - Word puzzles - Number puzzles - Visual puzzles - Games such as darts/bridge/dominoes - Playing musical instruments - New brain training technology - Computer games | 5%  2%  7.7%  7.1%  3.8%  1.1%  1.9%  4.5%  3.9% |
| Dos Santos 2015 | Use of medication can cause dementia | 25.4% |
| Luck 2012 | Brain/memory training can prevent dementia  Active life/participation can prevent dementia  Early detection/early diagnosis/medical therapy can prevent dementia  Scientific research can prevent dementia  Other strategies can prevent dementia | 47.4%  18.6%  3.8%  2.6%  1.3% |
| McParland 2012 | People who eat healthily and exercise regularly are less likely to get dementia | 29% |
| Shinan-Altman 2017 | Rated from 1 (strongly disagree) to 5 (strongly agree):  Perception that AD is attributable to psychological factors  Perception that AD is attributable to risk factors  Perception that AD is attributable to immunity | M=2.05 (SD=0.78)  M=2.47 (SD=0.65)  M=1.83 (SD=0.79) |
| Yang 2015 | Hypertension, diabetes, heart disease and brain injury are important risk factors for Alzheimer’s disease | 12.9% |

M=Mean

*Reverse scores used for pooling
