# Supplementary material for: What does the general public understand about prevention and treatment of dementia? A systematic review of population-based surveys
Source: PLoS One. 2018 Apr 19;13(4):e0196085. doi: 10.1371/journal.pone.0196085 (PMC5908164; doi:10.1371/journal.pone.0196085)
Supplement: S4 Table — M = Mean *Reverse scores used for pooling a59% ‘disagree’ or ‘strongly disagree’, 20% ‘don’t know’ b91% disagree, 2% uncertain. (DOCX) [file pone.0196085.s005.docx]

Table S4. Treatment results.

| Study first author, year | Phrase | % yes/agreed |
| --- | --- | --- |
| TREATMENT GENERAL | | |
| *People should seek help for memory problems* | | |
| Berwald 2016 | People should seek professional help for memory problems | 52% |
| Blendon 2012 | If exhibiting confusion and memory loss, you would go to a doctor to determine if cause was Alzheimer’s disease   - France - Germany - Poland - Spain - US | 88%  90%  85%  95%  89% |
| Dos Santos 2015 | If exhibiting confusion and memory loss, you would go to a doctor to determine if cause was Alzheimer’s disease | 94% |
| Yang 2015 | People with Alzheimer’s disease should seek professional help as soon as possible | 26% |
| *Believe that effective treatments for dementia exist* | | |
| Berwald 2016 | Feeling that nothing can be done | 12%* |
| Breining 2014 | Effective treatments exist | 28% |
| Diamond 2014 | Some types of dementia are treatable | 79% |
| Fowler 2015 | Belief that a treatment for Alzheimer’s disease is not currently available (agree or strongly agree) | 21%*^a^ |
| Leon 2015 | There are effective treatments against Alzheimer’s disease   - 2008 cohort - 2013 cohort | 28%  28% |
| Nguyen 2016 | Some types of dementia are treatable | 81% |
| Woo 2013 | Some types of dementia are treatable | 55% |
| Zheng 2016 | Some types of dementia are treatable | 54% |
| *Believe an effective treatment is available to slow progression of the disease* | | |
| Blendon 2012 | Believe an effective treatment currently available to slow the progression of the disease and make symptoms less severe   - France - Germany - Poland - Spain - US | 40%  42%  63%  27%  46% |
| Breining 2014 | Treatments can ease symptoms and improve the quality of life of patients suffering from Alzheimer’s disease | 84% |
| Dos Santos 2015 | Believe an effective treatment currently available to slow the progression of the disease | 42% |
| *Believe treatments are available to improve wellbeing* | | |
| Leon 2015 | There are treatments to improve the wellbeing of patients   - 2008 cohort - 2013 cohort | 84%  80% |
| *Believe that there is a cure for dementia* | | |
| Blendon 2012 | Alzheimer’s disease is fatal   - France - Germany - Poland - Spain - US | 44%*  33%*  34%*  42%*  61%* |
| Breining 2014 | Alzheimer’s disease is curable | 65% |
| Hudson 2012 | Alzheimer’s disease cannot be cured | 94%* |
| McParland 2012 | Dementia can be cured | 6% |
| Mi-Ra 2015 | Some types of dementia can be cured completely   - Males - Females | 37%  30% |
| Nielsen 2016 | Is there a cure?   - Danish - Polish - Turkish - Pakistani | 6%  21%  69%  45% |
| Park 2016 | Alzheimer’s disease is generally fatal | 36%* |
| Picco 2016 | With professional help, full recovery with no further problems is possible | 13% |
| Seo 2015 | Some types of dementia can be cured completely | 47.3% |
| Sun 2014 | In rare cases, people have recovered from Alzheimer’s disease | 16% |
| Yang 2015 | At present, Alzheimer’s disease can be cured | 39% |
| Zeng 2015 | We can cure patients of Alzheimer’s disease now | 62% |
| PHARMACOLOGICAL STRATEGIES | | |
| *Believe there are pharmacological treatments available* | | |
| Dos Santos 2015 | Pharmaceutical treatments useful for dementia | 90% |
| Riva 2012 | Do you know there are pharmacologic therapies available? | 64% |
| *Believe that medications can cure Alzheimer’s disease* | | |
| Ludecke 2016 | Prescription medications to cure Alzheimer’s disease are available | 9% |
| Park 2016 | Alzheimer’s disease can be cured with medication | 24% |
| *Believe that medications can help to treat dementia* | | |
| McParland 2012 | There are drug treatments that help with dementia | 75% |
| Mi-Ra 2015 | Drugs are useful in the treatment of dementia   - Males - Females | 79%  75% |
| Picco 2016 | Medicines prescribed by a psychiatrist considered helpful for someone with dementia | 84% |
| Riva 2012 | Do you think that pharmacological therapies are effective? | 58% |
| Seo 2015 | Drugs are useful in the treatment of dementia | 78.3% |
| *Specific medications helpful in the treatment of dementia* | | |
| Picco 2016 | Antibiotics considered helpful for someone with dementia  Sleeping pills considered helpful for someone with dementia  Antidepressants considered helpful for someone with dementia  Supplements considered helpful for someone with dementia  Tonics considered helpful for someone with dementia | 10%  21%  50%  48%  41% |
| NON-PHARMACOLOGICAL STRATEGIES | | |
| *Memory strategies* | | |
| Hudson 2012 | When a person has Alzheimer’s disease, using reminder notes is a crutch that can contribute to decline | 21% |
| Stites 2016 | When a person has Alzheimer’s disease, using reminder notes is a crutch that can contribute to decline | 26% |
| *Relaxation* | | |
| Picco 2016 | Course on relaxation or stress management helpful for someone with dementia | 76% |
| *Psychotherapy* | | |
| Hudson 2012 | People whose Alzheimer’s disease is not yet severe can benefit from psychotherapy for depression and anxiety | 71% |
| Picco 2016 | Counselling (in person) helpful for someone with dementia | 70% |
| Stites 2016 | People whose Alzheimer’s disease is not yet severe can benefit from psychotherapy for depression and anxiety | 76% |
| Sun 2014 | People whose Alzheimer’s disease is not yet severe can benefit from psychotherapy for depression and anxiety | 90% |
| *Social activity* | | |
| Picco 2016 | Getting out more, being social helpful for someone with dementia | 73% |
| Yang 2015 | There is no need for people with Alzheimer’s disease to participate in community activities | 6%*^b^ |
| *Good nutrition* | | |
| Hudson 2012 | Poor nutrition can make the symptoms of Alzheimer’s disease worse | 55% |
| Picco 2016 | Going on a special diet helpful for someone with dementia | 34% |
| Stites 2016 | Poor nutrition can make the symptoms of Alzheimer’s disease worse | 78% |
| Sun 2014 | Poor nutrition can make the symptoms of Alzheimer’s disease worse | 62% |
| *Natural treatments* | | |
| Berwald 2016 | Prefer to pursue alternative and natural treatments not supported by healthcare rather than seek a diagnosis | 18% |
| *Physical activity* | | |
| Picco 2016 | Becoming more physically active helpful for someone with dementia | 82% |
| *Educating the person with dementia* | | |
| Mi-Ra 2015 | Because a dementia patient does not have the ability to judge, the patient does not need to be given any explanation during treatment   - Males - Females | 65%  72% |
| Picco 2016 | Reading about how people have dealt with similar problems helpful for someone with dementia | 89% |
| *Cutting out alcohol* | | |
| Picco 2016 | Cutting out alcohol helpful for people with dementia | 62% |
| *Religious/spiritual methods* | | |
| Picco 2016 | Speaking with a religious advisor helpful for people with dementia | 45% |
| Berwald 2016 | Reliance on prayers and spiritual beliefs for a solution | 22% |
| HEALTH PROFESSIONALS | | |
| *General practitioner able to help treat dementia* | | |
| Dementia Australia 2017 | If I thought I had dementia I would visit my GP | 77% |
| Berwald 2016 | Belief that general practitioner will not be able to help | 28%* |
| Picco 2016 | Doctor or GP helpful for someone with dementia | 74% |
| *Other health professionals able to help treat dementia* | | |
| Picco 2016 | Psychiatrist helpful for someone with dementia  Psychologist helpful for someone with dementia  Social worker helpful for someone with dementia  Traditional Chinese medicine practitioner helpful for someone with dementia | 83%  74%  66%  29% |
| NOT POOLED | | |
| Dos Santos 2015 | Current treatments highly effective | 16% |
| Hailstone 2017 | I would want to go to see my doctor if I had memory problems: 1=strongly disagree, 7=strongly agree  Overall I think seeking help from my doctor for memory problems would be: 1=harmful, 7=beneficial  Overall, I think seeking help from my doctor for memory problems would be: 1=useless, 7=valuable  Overall I think seeking help from my doctor for memory problems would be: 1=bad, 7=good  My doctor would be able to provide treatments to help with memory problems: 1=strongly disagree, 7=strongly agree  My doctor would be able to tell me what services are available to help with memory problems: 1=strongly disagree, 7=strongly agree | Median = 6  (IQR 5.5-7)  Median = 7  (IQR 6-7)  Median = 7  (IQR 5-7)  Median = 7  (IQR 6-7)  Median = 6  (IQR 4-7)  Median = 6  (IQR 5.5-7) |
| Picco 2016 | Intervention considered helpful for someone with dementia   - Getting information from websites - Contacting an expert via email or a website - Yoga or meditation - Having an occasional drink to relax - Phone counselling - Close family - Close friends | 69%  67%  68%  10%  34%  84%  78% |

*Reverse scores used for pooling

^a^59% ‘disagree’ or ‘strongly disagree’, 20% ‘don’t know’

^b^91% disagree, 2% uncertain
