# Supplementary material for: What does the general public understand about prevention and treatment of dementia? A systematic review of population-based surveys
Source: PLoS One. 2018 Apr 19;13(4):e0196085. doi: 10.1371/journal.pone.0196085 (PMC5908164; doi:10.1371/journal.pone.0196085)
Supplement: S5 Table — Y = Yes, N = No, U = Unclear. (DOCX) [file pone.0196085.s006.docx]

Table S5. Results of quality assessment of peer-reviewed journal articles.

|  | *Is the sampling strategy relevant to address the quantitative research question?* | *Is the sample representative of the population under study?* | *Are measurements appropriate (clear origin, or validity known, or standard instrument)?* | *Is there an acceptable response rate (60% or above)?* |
| --- | --- | --- | --- | --- |
| Almeling et al (2014) | Y | Y | Y | N |
| Ayalon et al (2013) | Y | Y | Y | Y |
| Berwald et al (2016) | Y | Y | Y | U |
| Blendon et al (2012) | Y | U | Y | U |
| Bowes et al (2012) | Y | Y | Y | U |
| Breining et al (2014) | Y | Y | Y | N |
| Diamond et al (2014) | U | U | Y | Y |
| Fowler et al (2015) | Y | Y | Y | U |
| Hailstone et al (2017) | Y | Y | Y | U |
| Hudson et al (2012) | Y | U | Y | N |
| Leon et al (2015) | U | Y | Y | U |
| Luck et al (2012) | Y | Y | Y | N |
| Ludecke et al (2016) | Y | Y | Y | Y |
| McParland et al (2012) | Y | Y | Y | N |
| Nguyen et al (2016) | Y | Y | Y | U |
| Nielsen et al (2016) | Y | Y | Y | Y |
| Park et al (2016) | Y | U | Y | N |
| Picco et al (2016) | Y | Y | Y | Y |
| Riva et al (2012) | Y | Y | Y | U |
| Roberts et al (2014) | Y | Y | Y | Y |
| Seo et al (2015) | Y | Y | Y | Y |
| Shinan-Altman et al (2017) | U | Y | Y | U |
| Smith et al (2014) | Y | Y | Y | N |
| Stites et al (2016) | Y | Y | Y | N |
| Sun et al (2014) | Y | Y | Y | U |
| Tan et al (2012) | Y | Y | Y | Y |
| Woo et al (2013) | U | Y | Y | U |
| Yang et al (2015) | Y | Y | Y | U |
| Zeng et al (2015) | Y | Y | Y | U |
| Zheng et al (2016) | Y | Y | Y | Y |

Y=Yes, N=No, U=Unclear
